# Assessing geographical inequity in availability of hospital services under the state-funded universal health insurance scheme in Chhattisgarh state, India, using a composite vulnerability index

**DOI:** 10.1080/16549716.2018.1541220

**Published:** 2018-11-14

**Authors:** Sulakshana Nandi, Helen Schneider, Samir Garg

**Affiliations:** a School of Public Health, University of the Western Cape, Bellville, South Africa; b Public Health Resource Network, Chhattisgarh, Raipur, India; c School of Public Health, UWC/MRC Health Services to Systems Unit, University of the Western Cape, Bellville, South Africa; d State Health Resource Centre, Chhattisgarh, Raipur, India; e School of Health Systems Studies, Tata Institute of Social Sciences, Mumbai, India

**Keywords:** Health equity, vulnerable populations, health insurance, availability of health services, universal health coverage

## Abstract

**Background**: Countries are increasingly adopting health insurance schemes for achieving Universal Health Coverage. India’s state-funded health insurance scheme covers hospital care provided by ‘empanelled’ private and public hospitals.

**Objective**: This paper assesses geographical equity in availability of hospital services under the universal health insurance scheme in Chhattisgarh state.

**Methods**: The study makes use of district data from the insurance scheme and government surveys. Selected socio-economic indicators are combined to form a composite vulnerability index, which is used to rank and group the state’s 27 districts into tertiles, named as highest, middle and lowest vulnerability districts (HVDs, MVDs, LVDs). Indicators of hospital service availability under the scheme – insurance coverage, number of empanelled private/public hospitals, numbers and amounts of claims – are compared across districts and tertiles. Two measures of inequality, difference and ratio, are used to compare availability between tertiles.

**Results**: The study finds that there is a geographical pattern to vulnerability in Chhattisgarh state. Vulnerability increases with distance from the state’s centre towards the periphery. The highest vulnerability districts have the highest insurance coverage, but the lowest availability of empanelled hospitals (3.4 hospitals per 100,000 enrolled in HVDs, vs 8.2/100,000 enrolled in LVDs). While public sector hospitals are distributed equally, the distribution of private hospitals across tertiles is highly unequal, with higher availability in LVDs. The number of claims (per 100,000 enrolled) in the HVDs is 3.5-times less than that in the LVDs. The claim amounts show a similar pattern.

**Conclusions**: Although insurance coverage is higher in the more vulnerable districts, availability of hospital services is inversely proportional to vulnerability and, therefore, the need for these services. Equitable enrolment in health insurance schemes does not automatically translate into equitable access to healthcare, which is also dependent on availability and specific dynamics of service provision under the scheme.

## Background

Inequity in health refers to differences in health that are ‘systematic, socially produced (and therefore modifiable) and unfair’ ([1], p. 3). Such inequities emerge from a number of social, economic, political and geographical factors [1,2]. These factors often converge geographically in multi-dimensional forms of deprivation [2,3]. Such areas or populations typically also have less access to healthcare, despite being in greater need, exhibiting the phenomenon described as the ‘Inverse Care Law’ by Hart [4]. Moreover, historical factors and absorptive capacity often lead to an ‘infrastructure-inequality trap’ from which these areas find it very difficult to emerge [5].

In India, inequalities in healthcare availability and utilization exist along geographical (rural–urban), gender, class and caste lines, and are reflected in inequitable health outcomes [6–9]. As elsewhere, these patterns of inequality are often clustered geographically across states or within a state in areas [8]. As these inequalities can be regarded as both avoidable and unfair, we refer to them as ‘geographical inequities’.

The health system has a crucial role in addressing social inequity [1,10–12] and health equity is considered a central goal of Universal Health Coverage (UHC) [13,14]. Low- and middle-income countries (LMICs) are increasingly adopting state-funded health insurance schemes as a strategy for achieving UHC [15,16]. Many of these countries have mixed health systems and their insurance schemes have sought to include private hospitals as providers of healthcare, along with public hospitals [15]. However, evidence from India and other LMICs shows that there is a skewed distribution of health facilities and resources, with the formal private sector mostly concentrated in urban and richer areas and employing the majority of the medical specialists [8,16–21]. Studies have also found that insurance coverage is not a sufficient condition to ensure equity in healthcare utilization [22,23] and that non-financial factors, such as the nature, distribution and performance of health systems, are equally important for improved access [19,23–29]. Moreover, inequitable utilization tends to be clustered geographically [19,26,29]. This study seeks to further examine the phenomenon of geographical equity in availability of facilities under publicly-funded health insurance schemes, including the distribution of private and public hospitals.

In the last decade and a half, many Indian states have introduced publicly-financed health insurance schemes [30,31]. At the national level, the *Rashtriya Swasthya Bima Yojana* (RSBY) or the National Health Insurance Scheme, a state funded scheme for hospitalization, was introduced by the Government of India in 2007. The goal of RSBY/MSBY is greater financial risk protection for all, through choice of public or private provider [32,33]. RSBY has, thus, enabled large-scale state funding of private sector hospital care in India for the first time. Studies in India assessing the equity impact of the publicly-financed insurance schemes have mainly examined equity in insurance coverage. Lower enrolments have been found in remote rural areas, poorer districts, socio-economically vulnerable groups, indigenous communities and female-headed households [34–36]. A few studies of utilization of hospital care under state-funded health insurance in India have found inequities based on caste, economic status, education and urban–rural residence [36–38].

Where geographical equity has been studied, the focus has been on differences between urban and rural areas or between regions or states [34,36–39]. For example, Narayana [34] found inequitable distribution of empanelled hospitals, especially of private hospitals, within six Indian states.

Recognizing the multi-dimensional nature of vulnerability and its frequent geographical clustering, the aim of this study was to assess geographical inequity in availability of hospital services under the publicly-funded universal health insurance scheme in the state of Chhattisgarh [8,40].

Chhattisgarh is a useful case to examine for a number of reasons. It is one of the poorest states in India [41], with around one-third of its population belonging to indigenous tribal communities [42]. It has a state-funded health insurance scheme for hospital care that is universal in design [32] and the second highest rate of state-funded insurance enrollment in the country [43]. Further, the state has empanelled public and private sector hospitals to provide services under this scheme [32].

### State-funded universal health insurance scheme in Chhattisgarh

Chhattisgarh is one of the first states to start implementing RSBY in 2009. In 2012, the state expanded RSBY, meant for people living below the poverty line, to all families of the state through the *Mukhyamantri Swasthya Bima Yojana* (MSBY) or the Chief Minister’s Health Insurance Scheme [32]. This universal scheme is, thus, supposed to cover all families living in the state, regardless of income or nature or type of employment. Chhattisgarh has a total of 27 districts and the insurance scheme covers all of them. The benefit package is uniform for all enrolled, irrespective of economic status, employment or residence. The scheme allows for enrolment of a maximum of five members per household. Even if a household member is part of another scheme, they are still eligible for enrolment under RSBY/MSBY. RSBY and MSBY constitute the bulk (nearly 94%) of health insurance enrolment in the state [44]. Each household has to pay a one-time registration fee of Rs. 30 (USD 0.43 in August 2018), after which they are enrolled and provided a biometric smart card. The government (state and centre) pays the premium on behalf of the enrolled families. Currently there are 5.5 million active insurance cards in the state [32]. The premium is paid to an insurance company that is selected through a bidding process. The empanelled private and public hospitals are supposed to provide cashless services, based on pre-determined packages. The hospitals then claim the insurance amount as per the package used, from the insurance company [32]. At the time of the study, enrolled households were eligible for hospitalization costs of up to Indian Rupee (INR) 30,000 (USD 430 in August 2018) annually, which has since increased to an annual entitlement of INR 50,000 (USD 717 in August 2018). The scheme also provides for transport costs of INR 100 (USD 1.43 in August 2018) per hospitalization, up to a maximum of INR 1,000 (USD 14.33 in August 2018) annually [32].

Studies in Chhattisgarh by the authors and others have found that the more vulnerable groups, such as the poor, tribal communities, urban poor and those living in rural areas, are more likely to use the public sector for hospitalization, irrespective of their enrolment status [44–47]. In addition, out of pocket expenditure in the private sector is much higher than in government facilities, even with the use of insurance [44,45]. Practices of ‘cherry picking’ and selective provision of services have also been documented in the private sector under the insurance scheme [45,48,49].

Both the state and central governments of India are looking to expand the publicly-funded health insurance scheme. An analysis of the main budget heads of the Chhattisgarh state budget from financial years 2013–14 to 2017–18 shows that the budget for the universal health insurance scheme has increased, both in real terms and as a proportion of the total health budget [50]. Recently, the Government of India announced an expansion of state-funded health insurance with the intention to cover 100 million poor families with an annual insurance entitlement of half a million rupees per family [51].

### Aim

The aim of this paper is to assess geographical equity in the availability of hospital services under the state-funded universal health insurance scheme across Chhattisgarh state.

## Methods

### Conceptual framework

In this study, ‘availability’ is understood to be one dimension of the broader concept of access [52]. Access is defined as the ‘degree of fit’ between the health system and individuals, and communities, with respect to the dimensions of availability, affordability and acceptability [53]. Access creates the opportunity for utilization and the possibility for improved health outcomes [54]. Further, equity in access means the fair or equal distribution of health and healthcare [55].

**Figure 1.**
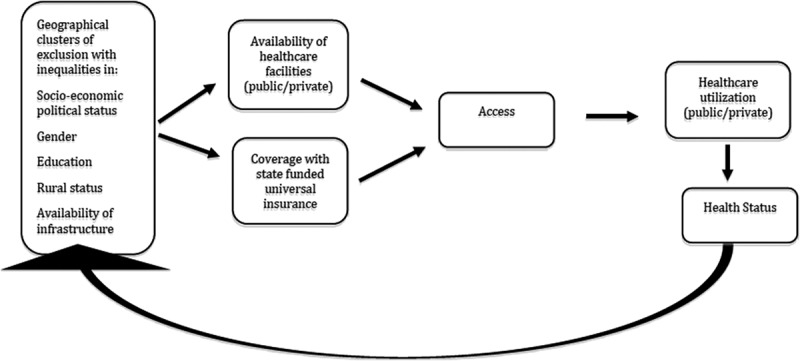
Conceptual framework illustrating pathways of equity under the universal health insurance scheme.

Figure 1 illustrates the pathways of health inequity explored by this study. Socio-economic status, gender, education, rural status and availability of infrastructure have been selected as indicators of inequity, which often converge geographically. The combination of insurance coverage and availability of hospitals enable access and, therefore, utilization and, ultimately, effective coverage and improved health. Although access and utilization depend on various factors, this analysis focuses specifically on the availability factor. Other dimensions of access, such as affordability, are reported elsewhere by the authors [44].

### Study design

Using secondary data, a cross-sectional, descriptive study of the relationship between geographical vulnerability, insurance coverage and hospital service availability under the universal health insurance scheme in Chhattisgarh state was conducted. Since the study seeks to study this across geographical areas of the entire state, all 27 districts were taken into consideration. First, the relationship between individual socio-economic or vulnerability indicators and health insurance indicators was examined across the 27 districts of the state. The districts were then ranked and grouped into tertiles and categorized as highest, middle and lowest vulnerability groups using a composite vulnerability index (VI). Finally, the indicators of hospital services availability under the universal health insurance scheme were mapped across the tertiles.

### Data sources

The study made use of district level data from a number of sources. District socio-economic indicators were obtained from various government surveys, detailed in the next section [42,56]. The district RSBY/MSBY programme data on empanelled hospitals, enrolment and claims for the financial year 2015–16 in Chhattisgarh were accessed from the State Department of Health and Family Welfare. The data used as inputs for the analysis are reported in Supplementary File 1.

### Vulnerability index

The methodology for measuring multi-dimensional vulnerability is well established in the literature [57–61]. However, global indices do not include certain factors, such as caste, which has been found to be a critical determinant of socio-economic vulnerability in India [7,8]. The Constitution of India has recognized disadvantaged social groups and categorized them into the ‘Scheduled Castes’, ‘Scheduled Tribes’, and ‘Other Backward Classes’. For this study, the authors, thus, developed an adapted composite vulnerability index by combining selected socio-economic or vulnerability indicators relevant to the Indian and state context. Specifically, the index includes indices related to the agricultural economy, caste, rural–urban divide, gender inequality and infrastructure availability. The indicators were, thus, selected based on their face validity in the Indian context, accepted practice in the literature, the availability of data for all 27 districts and reliability of the data source. The indicators and the rationale for their selection are presented in Table 1 [7,8,42,56,62–68].

**Table 1.** List of indicators selected for developing the vulnerability index, along with the rationale for their selection.

Name of indicator	Dimension	Rationale for selection	Source of data
Proportion of Scheduled Caste (SC) and Scheduled Tribe (ST) population	Social vulnerability	Caste or social group is considered as a critical determinant of socio-economic vulnerability in India [7,8]	Registrar General of India. GoI [42]
Proportion of un-irrigated net sown area	Economic vulnerability	Rural poverty is much lower in irrigated than in rainfed areas [62,63]	Directorate of Economics & Statistics. GoCG [56]
Female illiteracy	Education and gender inequality	Education and gender inequality are important indicators of vulnerability [64–66]	Registrar General of India. GoI [42]
Proportion of rural population	Rural status	Rural populations are more vulnerable than urban [8]	Registrar General of India. GoI [42]
Year of formation of the district	Availability of infrastructure	The older districts would have better health and other relevant infrastructure and administrative capacity than newer districts established in the last few years [67,68]. For instance, in the newer districts, the district hospitals are still under the process of being established	Directorate of Economics & Statistics. GoCG [56]

Global indices tend to take into account health status or health service utilization. However, the index developed for this study deliberately did not include these indicators, because health status and utilization can be an outcome of availability of services, as presented in the conceptual framework.

The index was computed using the United Nations Development Programme’s (UNDP) method for calculating the Human Development Index (HDI) [58], namely, as an unweighted average of normalized values of the five indicators in the index. The HDI method was used for this study, as a normalized indicator provides performance measure of a geographical unit in relation to the best and worst performance on that indicator. This is useful to rank the districts and, thereby, assess geographical equity. Following the UNDP method, the indicators were given equal weightage in the index. This is because, as mentioned above, the authors selected the indicators to highlight different kinds of vulnerability. All the dimensions were regarded as equally important, with no hierarchy imposed amongst them.

The method for computation was as follows: Indicators were normalized (brought into a common scale) using the formula Yi = (Xi – Xmin)/(Xmax – Xmin), where Yi is the normalized indicator for district i, Xi is the corresponding pre-normalization figure and Xmax and Xmin are the maximum and minimum values of the same indicator across all districts. The normalized indicator varies between 0 and 1 for all districts, with 0 being the least vulnerable and 1 being the most vulnerable. A simple addition of the normalized values for the five indicators forms the index of vulnerability. The calculations of the VI are presented in Supplementary File 2.

The 27 districts were ranked using the vulnerability index (VI) and grouped into vulnerability tertiles, named as highest vulnerability districts (HVDs), middle vulnerability districts (MVDs) and lowest vulnerability districts (LVDs). The VI scores of the tertiles ranged from 4.9–3.7 for the HVDs; 3.6–2.9 for MVDs and 2.9–0.2 for LVDs. Division into tertiles allowed comparison of availability and representation of its inequality through ratios and differences. Categorization into vulnerability tertiles and presenting the results through maps is also proposed as a method to better communicate the results and simplify assimilation of data by policy-makers, practitioners and community stakeholders [69].

The vulnerability index could not be validated statistically, as the authors worked on aggregate district level rather than individual data.

### Indicators of the health insurance scheme

The insurance scheme indicators are given in Table 2. Health insurance enrolment rate was computed per 100,000 population for each district and vulnerability group. Availability of hospital services was calculated as the number of empanelled (public and private) hospitals per 100,000 enrolled (Table 2). Further, in order to explore utilization (public and private), the claims (numbers and amount) under health insurance were similarly calculated and compared. The  above  indicators have been calculated as ‘per 100,000 enrolled’ in the districts and not on the total census population of the districts. This was done in order to make one dimension of availability, which is insurance coverage and opportunity to use insurance, equal for all groups. Although the primary purpose of the study was to assess the availability of hospitals, utilization was also examined to see whether it followed the pattern of availability.

**Table 2.** Indicators of the health insurance scheme.

Dimension	Indicators
Availability of empanelled hospitals	No. of hospitals empanelled per 100,000 persons enrolled
	No. of private hospitals empanelled per 100,000 persons enrolled
	No. of public hospitals empanelled per 100,000 persons enrolled
	Proportion of Private/Public hospitals empanelled to total empanelled
Enrolment	Proportion of census (2011) population enrolled
	Proportion of census (2011) Households enrolled
Number and amount of claims	Total no. of claims/amount of claims per 100,000 persons enrolled
	No. of claims/amount of claims by private providers per 100,000 persons enrolled
	No. of claims/amount of claims by public providers per 100,000 persons enrolled
	Proportion of number by Public/Private hospitals to total claims/amount of claims

The location of health services and hospitals is very important for access, especially for the poor, and, although the services may be available outside the district, either within the state or in adjoining states, cost and distance act as barriers to access [8,15,19,21]. Therefore, ‘claims made by hospitals in the district’ was used in calculating the outcome indicators, instead of ‘claims of people living in the district’ that would have also included claims made outside the district.

### Data analysis

First, the relationship between the individual socio-economic/vulnerability indicators plus combined VI and the hospital service availability indicators was examined, using bivariate Pearson’s correlation analyses across all 27 districts. Significance was assessed as the 0.05 level.

Second, a comparative analysis of the three VI groups was done with respect to insurance coverage, availability of hospitals and number of claims made in the districts, using two measures to describe inequality, difference and ratio [70]. Historically, these measures of equality have been widely used as their simplicity ‘makes them intuitive and easily understood’ ([70], p. 29).

The ratio shows the relative inequality between two groups and is calculated by dividing the value of one group by the other. This measure can be calculated only for pairwise comparisons [70]. Ratios between the lowest vulnerability district and highest vulnerability district groups and between the middle vulnerability district and highest vulnerability district groups were, thus, calculated for each indicator.

Finally, the 27 districts in the state were mapped by vulnerability tertiles and availability indicators through Geographical Information System (GIS) mapping. This was done using software called Map Window (version 5), an open source desktop GIS application [71]. The shape file of the map of Chhattisgarh with district boundaries marked was obtained from the state government. The first layering on the shape file involved showing the districts in three different shades according to their MVD-HVD-LVD status from a MS Excel file. Then districtwise indicators of availability of public and private facilities per 100,000 enrolled was superimposed on the map as the next layer. This method was used, as spatial differences can be better represented through visualization via maps.

### Ethics approval

As this study is based on secondary data, consent procedures were not required. Ethics approval for the research programme, of which this study forms one component, was obtained from the University of the Western Cape, where the first author is registered for a PhD.

## Results

### Vulnerability index and health insurance indicators across 27 districts

The correlation between individual vulnerability indicators and the insurance scheme indicators was studied using Pearson’s Correlation Coefficients (Supplementary File 3). It shows, amongst others, that the number of empanelled hospitals per 100,000 enrolled was negatively correlated with rural population and positively correlated with age of district, namely, the older the district, the higher the availability of hospitals.

The districtwise distribution of the VI and insurance scheme indicators, along with the classification of the districts into HVD, MVD and LVD, are presented in Table 3.

**Table 3.** Vulnerability Index and insurance scheme indicators across districts.

SN	District	VI	Vulnerability tertile	Insurance scheme indicators					
				Hospitals/100,000 enrolled population	Public hospital/100,000 enrolled	Private hospital/100,000 enrolled	No. of claims/100,000 enrolled population	No. of public claims/100,000 enrolled population	No. of private claims/100,000 enrolled population
1	Sukma	4.9	HVD	6.8	3.4	3.4	399	334	65
2	Bijapur	4.7	HVD	4.5	4.5	0.0	5628	5628	0
3	Narayanpur	4.5	HVD	7.3	4.9	2.4	6762	5052	1709
4	Kondagaon	4.3	HVD	4.8	2.9	1.9	3441	1247	2194
5	Balrampur	4.2	HVD	5.5	5.2	0.3	1718	1341	377
6	Dantewada	4.2	HVD	3.9	3.9	0.0	4053	4053	0
7	Jashpur	3.9	HVD	3.0	2.6	0.4	2672	1289	1383
8	Surajpur	3.8	HVD	1.3	1.1	0.1	937	893	44
9	Bastar	3.7	HVD	3.5	2.6	1.0	3492	1968	1524
10	Kanker	3.6	MVD	5.8	4.0	1.9	6841	3462	3379
11	Sarguja	3.4	MVD	5.6	3.0	2.6	7185	2427	4758
12	Gariyabandh	3.3	MVD	2.4	1.6	0.8	878	649	229
13	Kawardha	3.3	MVD	3.3	1.1	2.2	1890	161	1730
14	Koria	3.2	MVD	3.0	2.1	0.9	3001	2061	940
15	Mungeli	3.2	MVD	2.0	0.8	1.2	1215	25	1190
16	Korba	3.1	MVD	5.4	1.2	4.2	3928	872	3056
17	Mahasamund	3.0	MVD	2.7	0.8	1.9	2693	634	2059
18	Bemetara	2.9	MVD	1.5	1.2	0.3	933	575	358
19	Balodabazar	2.9	LVD	2.5	1.4	1.2	1583	1202	381
20	Raigarh	2.8	LVD	6.0	3.2	2.7	3148	686	2461
21	Rajnandgaon	2.7	LVD	8.5	5.2	3.3	5120	1327	3793
22	Balod	2.6	LVD	3.4	1.4	2.0	3882	841	3041
23	Janjgir	2.4	LVD	2.3	1.0	1.3	2590	937	1653
24	Dhamtari	2.1	LVD	9.0	4.7	4.3	12,849	2511	10,338
25	Bilaspur	1.7	LVD	11.6	1.5	10.1	15,000	1092	13,908
26	Durg	0.6	LVD	9.4	2.2	7.3	7428	1449	5978
27	Raipur	0.2	LVD	18.7	1.0	17.7	20,042	3136	16,906

The Pearson’s Correlation Coefficients between the combined vulnerability index and hospital availability indicators, with significance at the 0.05 level, are reported in Table 4. The findings show that the number of empanelled hospitals per 100,000 enrolled was negatively correlated with the vulnerability index. When compared by sector, availability of empanelled private hospitals was negatively correlated with the vulnerability index, while empanelled public hospitals showed a pro-vulnerability pattern.

**Table 4.** Correlation between the district vulnerability index and insurance scheme indicators (Pearson’s correlation coefficient with 95% confidence intervals).

	Vulnerability index			
			95% CI	
Availability indicators	Correlation coefficient	*p*- value	Lower limit	Upper limit
Enrolled population as proportion of census population	−0.077	0.702	−0.444	0.312
Enrolled HHs as proportion of census HHs	−0.133	0.508	−0.488	0.260
Number of empanelled hospitals/100,000 enrolled	−0.583	0.001*	−0.788	−0.260
Public empanelled hospitals/100,000 enrolled	0.414	0.032*	0.040	0.686
Private empanelled hospitals/100,000 enrolled	−0.750	< 0.001*	−0.879	−0.517
Number of claims/100,000 enrolled	−0.630	< 0.001*	−0.815	−0.328
Number of public claims/100,000 enrolled	0.197	0.324	−0.198	0.537
Number of private claims/100,000 enrolled	−0.760	< 0.001*	−0.884	−0.533

* Significant values at 0.05 level.

### Vulnerability tertiles

**Figure 2.**
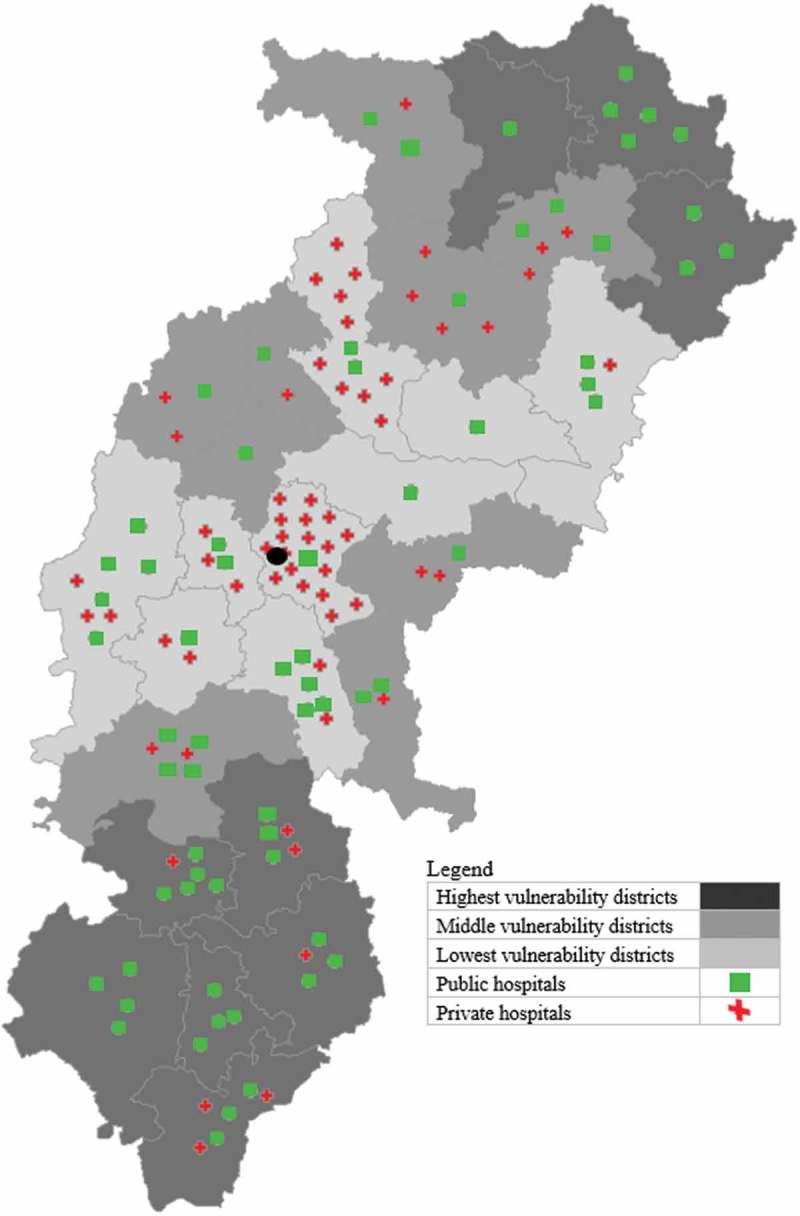
Chhattisgarh state map showing empanelled public and private hospitals per 100,000 enrolled across districts and vulnerability groups.

The map of the 27 districts in the state shows the distribution of the vulnerability index tertiles (Figure 2). The Highest Vulnerability Districts (HVDs) are the farthest (both north and south) from the state capital, Raipur (represented by the black dot). The Middle Vulnerability Districts (MVDs) are also in the periphery, while the Lower Vulnerability Districts (LVDs) consist of districts that are in the middle of the state and near to the state capital.

### Comparison of health insurance indicators across vulnerability groups

As of April 2016, 4.1 million households and 12.5 million persons had been enrolled in RSBY/MSBY. Enrolment was highest in the HVDs (52.5%), followed by MVDs (51.7%) and LVDs (46.1%).

In 2016, a total of 735 hospitals were empanelled under RSBY/MSBY in Chhattisgarh. Of these, 273 (37.1%) were public and 462 (62.9%) were private hospitals. The number of hospitals per 100,000 enrolled was highest in the LVDs (8.2) and lowest in the HVDs (3.4), followed by the MVDs (3.7) (Table 5). The availability of public hospitals was similar across the district groups, with slightly higher availability in HVDs (2.8 in HVDs, 1.8 in MVDs and 2.2 in LVDs per 100,000 enrolled). In contrast, there was variation in availability of private hospitals (6.0 in LVDs, 1.9 in MVDs and 0.6 in HVDs per 100,000 enrolled) (Table 5).

**Table 5.** Number of hospitals empanelled, number of claims and claim amount for the financial year 2015–16, disaggregated by public/private, according to vulnerability group.

VI Groups	Highest vulnerability districts (HVD)	Middle vulnerability districts (MVD)	Lowest vulnerability districts (LVD)	State total	Ratio between MVD and HVD	Ratio between LVD and HVD
Total number of empanelled hospitals (2016)	83	142	510	735		
Empanelled hospitals per 100,000 enrolled population	3.4	3.7	8.2	5.9	1.1	2.4
Empanelled public hospitals per 100,000 enrolled population	2.8	1.8	2.2	2.2	0.6	0.8
Empanelled private hospitals per 100,000 enrolled population	0.6	1.9	6	3.7	3.2	10
Total number of claims in 2015–16	59,432	133,237	516,104	708,773		
Total claims (nos) per 100,000 enrolled	2,399.7	3,480.3	8,341.8	5,673.9	1.5	3.5
Public claims (nos) per 100,000 enrolled	1,606.0	1,291.9	1,461.0	1,437.9	0.8	0.9
Private claims (nos) per 100,000 enrolled	793.7	2,188.4	6,880.9	4,235.9	2.8	8.7
Total claims amount (in INR million) in 2015–16	307	780	2,778	3,864.9		
Total claims amount (in INR 100,000) per 100,000 enrolled	124.1	203.6	449.0	309.4	1.6	3.6
Public claims amount (in INR 100,000) per 100,000 enrolled	70.3	49.1	48.3	52.9	0.7	0.7
Private claims amount (in INR 100,000) per 100,000 enrolled	53.9	154.6	400.6	256.5	2.9	7.4

Figure 2 also shows the distribution of empanelled public and private hospitals across the vulnerability tertiles per 100,000 enrolled. Hospitals, especially private hospitals, are concentrated in the LVDs, and in particular the capital Raipur, and the more urban districts of Bilaspur, Durg and Dhamtari.

The number of claims made by hospitals was also calculated across tertiles. The pattern of utilization was similar to hospital service availability (Table 5). There was a 3.5-fold difference in the number of claims per 100,000 enrolled in the HVDs (2,400) vs the LVDs (8,342) (Table 5). When disaggregated by sector, the number of claims (per 100,000 enrolled) made by the public sector across district groups was similar, in contrast to the number of claims (per 100,000 enrolled) made by the private hospitals. Claim numbers in the private sector in the LVDs were close to 9-times that of the HVDs (Table 5). Finally, the total claim amount in the financial year 2015–16 was INR 3864.9 million (USD 60 million) for the whole state, following the same patterns of inequality across vulnerability tertiles as claim numbers.

## Discussion

The study found a geographical pattern of vulnerability, increasing with distance from the centre (and the state capital) towards the periphery of the state. Indicators of socio-economic vulnerability converged geographically to form geographical clusters of inequity, reflecting the multi-dimensional and intersectional nature of deprivation [2,3,25].

When the districts were grouped into vulnerability tertiles, insurance enrolment tended towards a pro-poor pattern, with enrolment levels greater in the highest vulnerability districts.

However, on examining the availability of empanelled hospitals, patterns of inequality emerged among the vulnerability tertiles. The availability of hospitals under the insurance scheme was highly unequal among the tertiles. The geographical areas that were suffering from multiple vulnerabilities had poorer availability of hospital service. While the public sector hospitals were distributed equally, the distribution of the private hospitals across the vulnerability groups was highly unequal. Utilization (claim numbers and amounts) also followed a similar pattern to the availability of hospitals.

One of the main objectives of the RSBY/MSBY is that of giving people a ‘choice of provider’ [32,33]. The findings suggest that, in the more vulnerable areas, this ‘choice’ was limited. The findings on the concentration of hospitals, especially private hospitals in less vulnerable and more urbanized areas, is corroborated by studies in India and other LMICs [8,17,19,21].

Literature also shows that areas and populations with higher socio-economic vulnerability usually have the worst health indicators, and, therefore, have higher health needs [2,3,6–8,72]. The above pattern, thus, exhibits Hart’s [4] ‘Inverse Care Law’, where availability of health services is inversely correlated with need. The unequal resource allocations through claims under the state insurance scheme may also lead to a deepening of the ‘infrastructure inequality trap’ [5] in the state.

Those who have discussed the inherent limitations of insurance-like, demand-side interventions argue that there is a need to focus on and strengthen the supply side of health service provision if all aspects of access and equity are to be addressed [24,73,74], failing which, Universal Health Coverage would be ‘nominal’ rather than ‘effective’ ([28], pp. 26–27).

### Limitations

First, the vulnerability index (VI) could not be validated statistically, as the authors worked on aggregate data. However, the indicators were selected based on literature and theory and the index has face validity. The index is an improvement over a single indicator for determining vulnerability and indices measuring multi-dimensional vulnerability have been used by many [57–61]. Further, the VI was computed using a validated procedure used by the United Nations Development Programme (UNDP) for the Human Development Index (HDI) [58].

Second, data on the profile of services and size of the empanelled hospitals could not be accessed. Although these may vary, public and private hospitals are empanelled by the government under RSBY/MSBY on the basis of certain defined criteria like size of hospital. It is, therefore, assumed that the same parameters have been used by government in empanelling the hospitals in all districts.

Third, people with RSBY enrolment are eligible for using the insurance in other states that are implementing RSBY. However, it was not possible to find out whether anyone had accessed RSBY-based care in other states during 2015–16, as the data is not available from the Chhattisgarh state nodal office.

## Conclusion

The study finds that there is a geographical concentration of vulnerability. The study provides directions and tools for further research on the impact of geographical clustering of vulnerability on health equity. The availability of hospital services under the state-funded universal health insurance scheme in Chhattisgarh is unequal and inversely related to vulnerability and, thus, the need for these services. Although health insurance coverage was equitable, the availability of services was not. The study underlines the need for governments to make efforts to improve availability of services and ensure equitable distribution of hospital services in all areas and populations. Without appropriate policies for improving services availability in the more vulnerable areas, coverage with an insurance scheme is unlikely to achieve the equity goals of Universal Health Coverage (UHC).

## Paper context

Countries are increasingly choosing to implement state health insurance schemes for achieving Universal Health Coverage. This study finds that equitable enrolment in health insurance schemes does not automatically translate into equitable healthcare access. Dimensions of socio-economic vulnerability often converge geographically to form ‘clusters of inequity’ that have less availability of healthcare. Without improving health services availability in the more vulnerable areas, coverage with an insurance scheme is unlikely to achieve the equity goals of UHC.

## References

[1] Whitehead M, Dahlgren G. Levelling up (part 1): a discussion paper on concepts and principles for tackling social inequities in health [Internet]. Copenhagen: Conpenhagen WHO; 2006 Available from: http://www.who.int/social_determinants/resources/leveling_up_part1.pdf

[2] Mcintyre D, Muirhead D, Gilson L. Geographic patterns of deprivation in South Africa : informing health equity analyses and public resource allocation. Health Policy Plan. 2002;17:30–12. 12477739 10.1093/heapol/17.suppl_1.30

[3] Pratt B, Hyder AA How can health systems research reach the worst-off? A conceptual exploration. BMC Health Serv Res [Internet]. 2016;16 DOI:10.1186/s12913-016-1868-6 PMC5123377 28185590

[4] Hart JT The inverse care law. Lancet [Internet]. 1971 [cited 2018 5 31];297:405–412. Available from: https://www.sciencedirect.com/science/article/pii/S014067367192410X 10.1016/s0140-6736(71)92410-x 4100731

[5] Stuckler D, Basu S, McKee M Health care capacity and allocations among South Africa’s provinces: infrastructure-inequality traps after the end of apartheid. Am J Public Health [Internet]. 2011 [cited 2014 3 25];101:165–172. Available from: https://www.ncbi.nlm.nih.gov/pmc/articles/PMC3000713/pdf/165.pdf 10.2105/AJPH.2009.184895 PMC3000713 21148716

[6] Joe W, Mishra US, Navaneetham K Health inequality in India: evidence from NFHS 3. Econ Polit Wkly [Internet]. 2008;43:41–48. Available from: http://www.epw.in/special-articles/health-inequality-india-evidence-nfhs-3.html

[7] Baru R, Acharya A, Acharya S, et al Inequities in access to health services in India. Econ Polit Wkly. 2010;45:49–58.

[8] Balarajan Y, Selvaraj S, Subramanian SV India: towards universal health coverage 4 health care and equity in India. Lancet [Internet]. 2011;377:505–515. 10.1016/S0140-6736(10)61894-6 PMC3093249 21227492

[9] RGI Sample Registration System (SRS) bulletin volume 51, no. 1 [Internet]. 2017 [cited 2018 5 29] Available from: http://censusindia.gov.in/vital_statistics/SRS_Bulletins/SRS Bulletin -Sep_2017-Rate-2016.pdf

[10] Gilson L, Doherty J, Loewenson R, et al Challenging inequity through health systems: final report of the knowledge network on health systems [Internet]. 2007 Available from: http://www.who.int/social_determinants/resources/csdh_media/hskn_final_2007_en.pdf

[11] Mooney G, McIntyre D Why this book? In: McIntyre D, Mooney G, editors. Econ. Heal. Equity. Cambridge: Cambridge University Press; 2007 p. 3–10.

[12] Sen A Universal healthcare: the affordable dream. Harvard Public Heal Rev [Internet]. 2015;4 Available from: http://harvardpublichealthreview.org/universal-health-care-the-affordable-dream/

[13] WHO Making fair choices on the path to universal health coverage. Final report of the WHO consult- ative group on equity and universal health coverage [Internet]. Geneva; 2014 [cited 2014 5 20] Available from: http://apps.who.int/iris/bitstream/10665/112671/1/9789241507158_eng.pdf?ua=1

[14] Mcintyre D, Kutzin J. Health financing country diagnostic: a foundation for national strategy development [Internet]. Geneva: World Health Organization; 2016 Available from: http://apps.who.int/iris/bitstream/handle/10665/204283/9789241510110_eng.pdf;jsessionid=34150943047ADC9E7BF7095B10D64EC1?sequence=1

[15] Lagomarsino G, Garabrant A, Adyas A, et al Moving towards universal health coverage: health insurance reforms in nine developing countries in Africa and Asia. Lancet [Internet]. 2012 [cited 2014 3 24];380:933–943. Available from: http://www.sciencedirect.com/science/article/pii/S0140673612611477 10.1016/S0140-6736(12)61147-7 22959390

[16] Marten R, McIntyre D, Travassos C, et al An assessment of progress towards universal health coverage in Brazil, Russia, India, China, and South Africa (BRICS). Lancet (London, England) [Internet]. 2014 [cited 2015 9 17];384:2164–2171. Available from http://www.ncbi.nlm.nih.gov/pubmed/24793339 10.1016/S0140-6736(14)60075-1 PMC7134989 24793339

[17] Randive B, Chaturvedi S, Mistry N Contracting in specialists for emergency obstetric care- does it work in rural India? BMC Health Serv Res [Internet]. 2012;12:485 Available from: https://bmchealthservres.biomedcentral.com/articles/10.1186/1472-6963-12-485 10.1186/1472-6963-12-485 PMC3572412 23276148

[18] De Costa A, Al-Muniri A, Diwan VK, et al Where are healthcare providers? Exploring relationships between context and human resources for health Madhya Pradesh province, India. Health Policy (New York). 2009;84:269–276. 10.1016/j.healthpol.2009.03.015 19559495

[19] Macha J, Harris B, Garshong B, et al Factors influencing the burden of health care financing and the distribution of health care benefits in Ghana, Tanzania and South Africa. Health Policy Plan [Internet]. 2012;27:i46–i54. Available from: http://www.heapol.oxfordjournals.org/cgi/doi/10.1093/heapol/czs024 10.1093/heapol/czs024 22388500

[20] Jin C, Cheng J, Lu Y, et al Spatial inequity in access to healthcare facilities at a county level in a developing country: a case study of Deqing County, Zhejiang, China. Int J Equity Health. 2014;14(1):67. 26286033 10.1186/s12939-015-0195-6 PMC4545554

[21] Limwattananon S, Vongmongkol V, Prakongsai P, et al The equity impact of Universal Coverage : health care finance, catastrophic health expenditure, utilization and government subsidies in Thailand [Internet]. 2011 Available from: http://www.crehs.lshtm.ac.uk/thai_biafia_19jul.pdf

[22] Barraza-Lloréns M, Panopoulou G, Díaz BY Income-related inequalities and inequities in health and health care utilization in Mexico, 2000–2006. Rev Panam Salud Pública [Internet]. 2013;33:122–130. Available from: http://www.ncbi.nlm.nih.gov/pubmed/23525342 23525342 10.1590/s1020-49892013000200007

[23] Bennett S, Gilson L Health financing: designing and implementing pro-poor policies. DFID Heal Syst Resour Cent [Internet]. 2001;44:1–22. Available from: http://www.heart-resources.org/wp-content/uploads/2012/10/Health-financing.pdf

[24] Jacobs B, Ir P, Bigdeli M, et al Addressing access barriers to health services: an analytical framework for selecting appropriate interventions in low-income Asian countries. Heal Policy Plannning. 2012;27:288–300. 10.1093/heapol/czr038 21565939

[25] Meng Q, Xu L, Zhang Y, et al Trends in access to health services and financial protection in China between 2003 and 2011: A cross-sectional study. Lancet [Internet]. 2012;379:805–814. 10.1016/S0140-6736(12)60278-5 22386034

[26] Grogger J, Arnold T, Leon A, et al Heterogeneity in the effect of public health insurance on catastrophic out-of-pocket health expenditures: the case of Mexico. Health Policy Plan [Internet]. 2014 [cited 2014 7 7]:1–7. Available from: http://heapol.oxfordjournals.org/content/early/2014/06/12/heapol.czu037.full.pdf 10.1093/heapol/czu037 24924422

[27] Kusi A, Enemark U, Hansen KS, et al Refusal to enrol in Ghana’s national health insurance scheme: is affordability the problem? Int J Equity Health [Internet]. 2015 [cited 2015 12 5];14:2 Available from: https://www.ncbi.nlm.nih.gov/pmc/articles/PMC4300159/pdf/12939_2014_Article_130.pdf 10.1186/s12939-014-0130-2 PMC4300159 25595036

[28] Roberts MJ, Hsiao WC, Reich MR Disaggregating the universal coverage cube : putting equity in the picture. Heal Syst Reform. 2015;1:22–27. 10.1080/23288604.2014.995981 31519090

[29] Fenny AP, Kusi A, Arhinful DK, et al Factors contributing to low uptake and renewal of health insurance: a qualitative study in Ghana. Glob Heal Res Policy [Internet]. 2016;1:18 Available from: http://ghrp.biomedcentral.com/articles/10.1186/s41256-016-0018-3 10.1186/s41256-016-0018-3 PMC5693548 29202066

[30] Mackintosh M, Channon A, Karan A, et al Series UHC : markets, profi t, and the public good 1 What is the private sector? Understanding private provision in the health systems of low-income and middle-income. Lancet [Internet]. 2016;6736:1–10. 10.1016/S0140-6736(16)00342-1 27358253

[31] Public Health Foundation of India A critical assessment of the existing health insurance models in India [Internet]. New Delhi; 2011 Available from: http://planningcommission.nic.in/reports/sereport/ser/ser_heal1305.pdf

[32] Government of Chhattisgarh Website of RSBY and MSBY. Health and family welfare department government of Chhattisgarh [Internet]. 2018 [cited 2018 5 29] Available from: http://cg.nic.in/healthrsby/

[33] Government of India G. RSBY: RashtriyaSwasthya Bima Yojna [Internet]. 2017 [cited 2018 5 29] Available from: http://www.rsby.gov.in/

[34] Narayana D Review of the Rashtriya Swasthya Bima Yojana. Econ Polit Wkly. 2010;45:13–18.

[35] Rathi P. Evaluation of Rashtriya Swasthya Bima Yojana (RSBY): evaluation of Rashtriya Swasthya Bima Yojana (RSBY): a case study of Amravati district [Internet]. Bangalore; 2012 [cited 2018 5 29] Available from: http://www.iimb.ac.in/sites/default/files/u181/IIMB PGPPM Policy Folio_Paper_Prateek Rathi_March 2012.pdf

[36] Rao M, Katyal A, Singh PV, et al Changes in addressing inequalities in access to hospital care in Andhra Pradesh and Maharashtra states of India: a difference-in-differences study using repeated cross-sectional surveys. BMJ Open [Internet]. 2014 [cited 2015 9 10];4:e004471 Available from: https://bmjopen.bmj.com/content/4/6/e004471 10.1136/bmjopen-2013-004471 PMC4054646 24898084

[37] Prinja S, Chauhan AS, Karan A, et al Impact of publicly financed health insurance schemes on healthcare utilization and financial risk protection in India: a systematic review. PLoS One [Internet]. 2017;12:e0170996 Available from: http://dx.plos.org/10.1371/journal.pone.0170996 10.1371/journal.pone.0170996 PMC5289511 28151946

[38] Ranjan A, Dixit P, Mukhopadhyay I, et al Effectiveness of government strategies for financial protection against costs of hospitalization care in India. BMC Public Health [Internet]. 2018 [cited 2018 7 2];18 Available from: https://www.ncbi.nlm.nih.gov/pmc/articles/PMC5902925/pdf/12889_2018_Article_5431.pdf 10.1186/s12889-018-5431-8 PMC5902925 29661233

[39] Ghosh S Publicly-financed health insurance for the poor: understanding RSBY in Maharashtra. Econ Polit Wkly. 2014;49:93–99.

[40] Larson E, George A, Morgan R, et al 10 best resources on… intersectionality with an emphasis on low- and middle-income countries. Health Policy Plan. 2016;31:964–969. 27122486 10.1093/heapol/czw020

[41] World Bank Group Chhattisgarh poverty, growth & inequality [Internet]. 2016 [cited 2018 8 19] Available from: http://documents.worldbank.org/curated/en/166551468194958356/pdf/105848-BRI-P157572-PUBLIC-Chhattisgarh-Proverty.pdf

[42] Office of the registrar general & census commissioner I. Census of India website [Internet]. 2011 [cited 2018 5 29] Available from: http://censusindia.gov.in/2011-Common/CensusData2011.html

[43] International Institute for Population Sciences (IIPS) and ICF National Family Health Survey (NFHS-4), 2015–16. Mumbai, India: IIPS; 2017.

[44] Nandi S, Schneider H, Dixit P Hospital utilization and out of pocket expenditure in public and private sectors under the universal government health insurance scheme in Chhattisgarh state, India: lessons for universal health coverage. PLoS One [Internet]. 2017;12 Available from: http://dx.plos.org/10.1371/journal.pone.0187904 10.1371/journal.pone.0187904 PMC5693461 29149181

[45] Nandi S, Dasgupta R, Garg S, et al Uncovering coverage: utilisation of the universal health insurance scheme, Chhattisgarh by women in slums of Raipur. Indian J Gend Stud [Internet]. 2016;23:43–68. Available from: http://ijg.sagepub.com/cgi/doi/10.1177/0971521515612863

[46] Nandi S, Kanungo K, Khan H, et al A study of Rashtriya Swasthya Bima Yojana in. BMC Proc [Internet]. 2012;6:O5 Available from: http://www.biomedcentral.com/1753-6561/6/S1/O5

[47] Kundu D, Sharma N, Chadha S, et al Analysis of multi drug resistant tuberculosis (MDR-TB) financial protection policy: MDR- TB health insurance schemes, in Chhattisgarh state, India. Health Econ Rev [Internet]. 2018 [cited 2018 7 2];8:3 Available from: https://www.ncbi.nlm.nih.gov/pmc/articles/PMC5787110/pdf/13561_2018_Article_187.pdf 10.1186/s13561-018-0187-5 PMC5787110 29374822

[48] Dasgupta R, Nandi S, Kanungo K, et al What the good doctor said: a critical examination of design issues of the RSBY through provider perspectives in Chhattisgarh, India. Soc Change. 2013;43:227–243.

[49] CTRD Final report on evaluation of Rashtriya Swasthya Bima Yojana scheme in Chhattisgarh. [Internet]. Raipur; 2012 Available from: http://rsbychhattisgarh.in/WebSite/UploadDoc/70.pdf

[50] Department of finance GoCG G of C. Budget estimate, revised estimate and actual expenditure [Internet]. 2016 [cited 2018 5 29] Available from: http://finance.cg.gov.in/Budget_data/Yearwise7_object.asp

[51] Chatterjee P National health protection scheme revealed in India. Lancet (London, England) [Internet]. 2018;391:523–524. 10.1016/S0140-6736(18)30241-1 29617227

[52] Thiede M, Akweongo P, McIntyre D Exploring the dimensions of access In: McIntyre D, Mooney G, editors. Econ. Heal. Equity. Cambridge: Cambridge University Press; 2007 p. 103–123.

[53] McIntyre D, Thiede M, Birch S. Access as a policy-relevant concept in low- and middle-income countries. Health Econ. Policy. Law [Internet]. 2009 [cited 2014 6 21];4:179–193. Available from: http://www.ncbi.nlm.nih.gov/pubmed/19187569 10.1017/S1744133109004836 19187569

[54] REACH Collaboration. Researching Equity in Access to Health Care (REACH). Final technical report. Johannesburg, Cape Town, Hamilton (Ontario, Canada): Universities of Cape Town Witwatersrand & McMaster; 2012

[55] Rannan-Eliya R, Somanathan A Equity in health and health care systems in Asia In Ja M, editor. Elgar Companion to Heal. Econ. [Internet]. 2016 [cited 2014 5 18] Available from: http://www.equitap.org/publications/docs/Edward2006.pdf

[56] Government of Chhattisgarh. Directorate of Economics and Statistics, Chhattisgarh. [Internet]. Econ. Surv. 2014–15 2015 [cited 2018 5 29] Available from: http://descg.gov.in/

[57] Alkire S, Robles G. OPHI briefing 47 | Global multidimensional poverty index 2017 [Internet]. 2017 cited 2018 8 20] Available from: www.ophi.org.uk

[58] UNDP. Technical notes: Human Development Report 2015 [Internet] New York: United Nations Development Programme; 2015 Available from: http://hdr.undp.org/sites/default/files/hdr2015_technical_notes.pdf.

[59] Statistics South Africa The South African MPI [Internet]. Pretoria; 2014 Available from: http://www.statssa.gov.za/publications/Report-03-10-08/Report-03-10-082014.pdf

[60] Hosseinpoor AR, Bergen N, Barros AJD, et al Monitoring subnational regional inequalities in health: measurement approaches and challenges. Int J Equity Health [Internet]. 2016;15:18 Available from: http://equityhealthj.biomedcentral.com/articles/10.1186/s12939-016-0307-y 10.1186/s12939-016-0307-y PMC4730638 26822991

[61] United Nations Transforming our world: the 2030 agenda for sustainable development. general assembly 70 session [Internet]. 2015 p. 1–35. Available from: http://www.un.org/ga/search/view_doc.asp?symbol=A/RES/70/1&Lang=E

[62] Beero SK, Narayanamoorthy A Rural poverty and irrigation performance in India: a district-level study. Int J Soc Sci. 2014;3:329–345.

[63] Sharma RL, Abraham S, Bhagat R, et al Comparative performance of integrated farming system models in Gariyaband region under rainfed and irrigated conditions. Indian J Agric Res. 2017;51:64–68.

[64] Raj A Gender equity and universal health coverage in India. Lancet [Internet]. 2011 [cited 2018 5 29];377:618–619. Available from: https://www.thelancet.com/pdfs/journals/lancet/PIIS0140-6736(10)62112-5.pdf 10.1016/S0140-6736(10)62112-5 21227498

[65] Drèze J, Sen A An uncertain glory: India and its contradictions. New Delhi: Penguin Books; 2013.

[66] Vaid D. Gendered inequality in educational transitions. Econ. Polit. Wkly. [Internet]. 2004 [cited 2018 5 29];39:3927–3938. Available from: https://www.epw.in/journal/2004/35/special-articles/gendered-inequality-educational-transitions.html

[67] Tillin L Remapping India: new states and their political origins. New Delhi and New York: Oxford University Press; 2013.

[68] Chotia V, Rao NVM Examining the interlinkages between regional infrastructure disparities, economic growth, and poverty: A case of Indian States. Econ Ann. 2015;60:53–71.

[69] Dagenais C, Ridde V Policy brief as a knowledge transfer tool: to “make a splash”, your policy brief must first be read. Gac Sanit [Internet]. 2018 [cited 2018 8 28];32:203–205. Available from: https://www.sciencedirect.com/science/article/pii/S0213911118300360#! 10.1016/j.gaceta.2018.02.003 29609974

[70] WHO Handbook on health inequality monitoring: with a special focus on low- and middle-income countries [Internet]. Geneva: World Health Organisation; 2013 [cited 2018 5 29] Available from: http://apps.who.int/iris/bitstream/handle/10665/85345/9789241548632_eng.pdf;jsessionid=D5C1A0B9B1E296D03894744619557D1B?sequence=1

[71] The MapWindow Project [Internet] 2018 [cited 2018 8 22] Available from: https://www.mapwindow.org/

[72] Frenz P, Vega J Universal health coverage with equity: what we know, don’t know and need to know. Background paper for the First Global Symposium on Health Systems Research. Montreux, Switzerland; Nov 16–19; 2010.

[73] Mcintyre D Learning from experience: health care financing in low- and middle-income countries. Geneva: Global Forum for Health Research; 2007.

[74] Hooda SK Health insurance, health access and financial risk protection. Econ Polit Wkly. 2015;50:63–72.

