# Supplementary material for: Assessing geographical inequity in availability of hospital services under the state-funded universal health insurance scheme in Chhattisgarh state, India, using a composite vulnerability index
Source: Glob Health Action. 2018 Nov 14;11(1):1541220. doi: 10.1080/16549716.2018.1541220 (PMC6237177; doi:10.1080/16549716.2018.1541220)
Supplement: Supplemental Material [file ZGHA_A_1541220_SM0767.zip › Additional File 2_rev2.docx]

Additional File 2: Calculation of the Vulnerability Index

| SN | **District** | **Social vulnerability** | | **Economic vulnerability** | | **Gender/education** | | **Rural status** | | **Availability of infrastructure** | | **VI** |
| --- | --- | --- | --- | --- | --- | --- | --- | --- | --- | --- | --- | --- |
|  |  | **% SC & ST** | **Score** | **% Non-irrigated agriculture** | **Score** | **Female Illiteracy %** | **Score** | **Rural population %** | **Score** | **Years since formation of district** | **Score** | **Combined score** |
|  | **max value** | **85** |  | **100** |  | **78** |  | **95** |  | **5** |  |  |
|  | **min value** | **20** |  | **14** |  | **34** |  | **36** |  | **163** |  |  |
| 1 | BALOD | 40 | 0.30 | 49 | 0.41 | 37 | 0.06 | 87 | 0.86 | 5 | 1.00 | 2.6 |
| 2 | BALODABAZAR | 36 | 0.25 | 52 | 0.44 | 50 | 0.36 | 87 | 0.87 | 5 | 1.00 | 2.9 |
| 3 | BALRAMPUR | 67 | 0.73 | 89 | 0.87 | 60 | 0.60 | 95 | 1.00 | 5 | 1.00 | 4.2 |
| 4 | BASTAR | 64 | 0.68 | 97 | 0.97 | 63 | 0.66 | 84 | 0.81 | 70 | 0.59 | 3.7 |
| 5 | BEMETARA | 23 | 0.04 | 66 | 0.60 | 50 | 0.37 | 91 | 0.92 | 5 | 1.00 | 2.9 |
| 6 | BIJAPUR | 84 | 0.99 | 95 | 0.94 | 74 | 0.91 | 88 | 0.88 | 10 | 0.97 | 4.7 |
| 7 | BILASPUR | 40 | 0.31 | 57 | 0.50 | 47 | 0.29 | 69 | 0.55 | 156 | 0.04 | 1.7 |
| 8 | DANTEWADA | 75 | 0.85 | 100 | 1.00 | 67 | 0.76 | 76 | 0.68 | 19 | 0.91 | 4.2 |
| 9 | DHAMTARI | 33 | 0.20 | 24 | 0.12 | 40 | 0.12 | 81 | 0.77 | 19 | 0.91 | 2.1 |
| 10 | DURG | 20 | 0.00 | 37 | 0.27 | 34 | 0.00 | 36 | 0.00 | 111 | 0.33 | 0.6 |
| 11 | GARIYABANDH | 46 | 0.41 | 59 | 0.52 | 51 | 0.39 | 93 | 0.97 | 5 | 1.00 | 3.3 |
| 12 | JANJGIR | 36 | 0.25 | 22 | 0.09 | 47 | 0.30 | 86 | 0.85 | 19 | 0.91 | 2.4 |
| 13 | JASHPUR | 68 | 0.74 | 96 | 0.95 | 50 | 0.36 | 91 | 0.93 | 19 | 0.91 | 3.9 |
| 14 | KANKER | 60 | 0.61 | 86 | 0.84 | 47 | 0.30 | 90 | 0.91 | 19 | 0.91 | 3.6 |
| 15 | KAWARDHA | 35 | 0.23 | 68 | 0.63 | 60 | 0.58 | 89 | 0.90 | 19 | 0.91 | 3.3 |
| 16 | KONDAGAON | 75 | 0.85 | 96 | 0.95 | 62 | 0.63 | 90 | 0.91 | 5 | 1.00 | 4.3 |
| 17 | KORBA | 51 | 0.48 | 93 | 0.92 | 47 | 0.29 | 63 | 0.46 | 19 | 0.91 | 3.1 |
| 18 | KORIA | 54 | 0.53 | 92 | 0.91 | 48 | 0.32 | 69 | 0.56 | 19 | 0.91 | 3.2 |
| 19 | MAHASAMUND | 41 | 0.32 | 64 | 0.58 | 47 | 0.30 | 88 | 0.88 | 19 | 0.91 | 3.0 |
| 20 | MUNGELI | 38 | 0.28 | 52 | 0.44 | 57 | 0.52 | 91 | 0.92 | 5 | 1.00 | 3.2 |
| 21 | NARAYANPUR | 81 | 0.94 | 99 | 0.99 | 67 | 0.74 | 84 | 0.81 | 10 | 0.97 | 4.5 |
| 22 | RAIGARH | 49 | 0.45 | 77 | 0.73 | 45 | 0.25 | 84 | 0.80 | 70 | 0.59 | 2.8 |
| 23 | RAIPUR | 21 | 0.01 | 14 | 0.00 | 37 | 0.08 | 41 | 0.09 | 163 | 0.00 | 0.2 |
| 24 | RAJNANDGAON | 37 | 0.25 | 77 | 0.73 | 42 | 0.19 | 82 | 0.78 | 44 | 0.75 | 2.7 |
| 25 | SARGUJA | 62 | 0.65 | 90 | 0.88 | 56 | 0.50 | 84 | 0.81 | 70 | 0.59 | 3.4 |
| 26 | SUKMA | 85 | 1.00 | 99 | 0.99 | 78 | 1.00 | 89 | 0.89 | 5 | 1.00 | 4.9 |
| 27 | SURAJPUR | 51 | 0.48 | 87 | 0.85 | 57 | 0.53 | 91 | 0.93 | 5 | 1.00 | 3.8 |
