# Supplementary material for: Assessing geographical inequity in availability of hospital services under the state-funded universal health insurance scheme in Chhattisgarh state, India, using a composite vulnerability index
Source: Glob Health Action. 2018 Nov 14;11(1):1541220. doi: 10.1080/16549716.2018.1541220 (PMC6237177; doi:10.1080/16549716.2018.1541220)
Supplement: Supplemental Material [file ZGHA_A_1541220_SM0767.zip › Additional File 3_rev2.docx]

Additional File 3: Correlation (Pearson’s coefficient, 95% CI) between individual socio-economic indicators and insurance scheme indicators

| Insurance scheme indicators | | Socio-economic indicators | | | | |
| --- | --- | --- | --- | --- | --- | --- |
|  |  | Proportion of SC & ST | Proportion of non-irrigated agricultural land | Proportion of female illiteracy | Proportion of rural population | Years since district formation |
| Enrolled population as proportion of census population | Correlation coefficient | -0.193 | -0.077 | -0.344 | 0.160 | -0.163 |
|  | P value | 0.336 | 0.702 | 0.079 | 0.423 | 0.418 |
|  | Confidence interval | -0.534 - 0.202 | -0.444 - 0.312 | -0.640 - 0.042 | -0.234 - 0.509 | -0.515 - 0.227 |
| Enrolled HHs as proportion of census HHs | Correlation coefficient | -0.264 | -0.178 | -0.467* | 0.208 | -0.211 |
|  | P value | 0.183 | 0.375 | ***0.014*** | 0.298 | 0.291 |
|  | Confidence interval | -0.585 - 0.129 | -0.523 - 0.217 | -0.719 - -0.105 | -0.187 - 0.545 | -0.547 - 0.184 |
| No. of empanelled hospitals/100, 000 enrolled | Correlation coefficient | -0.214 | -0.367 | -0.321 | -0.700* | 0.787* |
|  | P value | 0.284 | 0.060 | 0.102 | ***<0.001*** | ***<0.001*** |
|  | Confidence interval | -0.549 - 0.181 | -0.655 - 0.016 | -0.625 - 0.068 | -0.853 - -0.435 | 0.58  - 0.898 |
| Public empanelled hospitals/100,000 enrolled | Correlation coefficient | 0.561* | 0.425* | 0.323 | 0.176 | -0.146 |
|  | P value | ***0.020*** | ***0.027*** | 0.100 | 0.381 | 0.467 |
|  | Confidence interval | 0.229 - 0.775 | 0.053 - 0.693 | -0.065 - 0.626 | -0.219 - 0.521 | -0.498 - 0.248 |
| Private empanelled hospitals/100,000 enrolled | Correlation coefficient | -0.438* | -0.537* | -0.450* | -0.773* | 0.847* |
|  | P value | ***0.022*** | ***0.004*** | ***0.018*** | ***<0.001*** | ***<0.001*** |
|  | Confidence interval | -0.701 - -0.069 | -0.761 - -0.197 | -0.709 - -0.084 | -0.891 - -0.556 | 0.688 - 0.928 |
| No. of claims/100,000 enrolled | Correlation coefficient | -0.277 | -0.456* | -0.402* | -0.644* | 0.779* |
|  | P value | 0.161 | ***0.017*** | ***0.038*** | ***<0.001*** | ***<0.001*** |
|  | Confidence interval | -0.594 - 0.115 | -0.712 - -0.091 | -0.678 - -0.025 | -0.822 - -0.349 | 0.566 - 0.894 |
| No. of public claims/100,000 enrolled | Correlation coefficient | 0.458* | 0.235 | 0.282 | -0.167 | 0.083 |
|  | P value | ***0.016*** | 0.238 | 0.154 | 0.404 | 0.680 |
|  | Confidence interval | 0.094 - 0.714 | -0.159 - 0.564 | -0.11 - 0.598 | -0.514 - 0.228 | -0.307 - 0.449 |
| No. of private claims/100,000 enrolled | Correlation coefficient | -0.461* | -0.580* | -0.537* | -0.648* | 0.824* |
|  | P value | ***0.015*** | ***0.002*** | ***0.003*** | ***<0.001*** | ***<0.001*** |
|  | Confidence interval | -0.715 - -0.098 | -0.786 - -0.256 | -0.761 -  -0.197 | -0.825  -  -0.355 | 0.646 -  0.917 |

* Significant at 0.05 level; significant values indicated in bold italics
